# Melatonin Enhances Cold Tolerance by Regulating Energy and Proline Metabolism in Litchi Fruit

**DOI:** 10.3390/foods9040454

**Published:** 2020-04-08

**Authors:** Gangshuai Liu, Yuxin Zhang, Ze Yun, Meijiao Hu, Jialiang Liu, Yueming Jiang, Zhengke Zhang

**Affiliations:** 1 College of Food Science and Engineering, Hainan University, Haikou 570228, China; lgsliugangshuai@163.com (G.L.); beakyhzhangyuxin@163.com (Y.Z.); jialiangliu0722@163.com (J.L.); 2 College of Food Science and Nutritional Engineering, China Agricultural University, Beijing 100083, China; 3 South China Botanical Garden, Chinese Academy of Sciences, Guangzhou 510650, China; yunze@scbg.ac.cn (Z.Y.); ymjiang@scbg.ac.cn (Y.J.); 4 Environment and Plant Protection Institute, Chinese Academy of Tropical Agricultural Sciences, Haikou 571101, China; humeijiao320@163.com

**Keywords:** litchi, chilling injury, melatonin, energy status, proline metabolism

## Abstract

Melatonin (MLT) is a vital signaling molecule that regulates multiple physiological processes in higher plants. In the current study, the role of MLT in regulating chilling tolerance and its possible mechanisms in litchi fruit during storage at ambient temperatures after its removal from refrigeration was investigated. The results show that the application of MLT (400 μM, dipping for 20 min) to ‘Baitangying’ litchi fruit effectively delayed the development of chilling injury (CI) while inhibiting pericarp discoloration, as indicated by higher chromacity values (L*, a*, b*) and anthocyanin levels. MLT treatment suppressed the enhancements of the relative electrical conductivity (REC) and malondialdehyde (MDA) content, which might contribute to the maintenance of membrane integrity in litchi fruit. MLT treatment slowed the decline in cellular energy level, as evidenced by higher adenosine triphosphate (ATP) content and a higher energy charge (EC), which might be ascribed to the increased activities of enzymes associated with energy metabolism including H^+^-ATPase, Ca^2+^-ATPase, succinate dehydrogenase (SDH), and cytochrome C oxidase (CCO). In addition, MLT treatment resulted in enhanced proline accumulation, which was likely a consequence of the increased activities of ornithine-δ-aminotransferase (OAT) and Δ^1^-pyrroline-5-carboxylate synthase (P5CS) and the suppressed activity of proline dehydrogenase (PDH). These results suggest that the enhanced chilling tolerance of litchi fruit after MLT treatment might involve the regulation of energy and proline metabolism.

## 1. Introduction

The fruit of litchi (*Litchi chinensis* Sonn.) plants are important tropical and subtropical crops with high commercial value due to their intriguing red pericarp, delicious taste, and abundant nutritional content [1]. Litchi is classified as a non-climacteric fruit whose quality deteriorates rapidly after harvest, as manifested by pericarp browning, anthocyanin degradation, loss of mass, reduced flavor and increased susceptibility to pathogens, which significantly restricts the supply chain of commodities [2]. Refrigeration, one of the most common storage strategies, can prolong the storage life of litchi fruit by a few weeks, depending on the specific cultivars and storage temperatures [3]. Nevertheless, some difficulties exist concerning the refrigeration of litchi because fruit quality can be subjected to gradual reduction during cold storage and undergo severe deterioration within hours once the chilled fruit are transferred to shelf conditions at ambient temperatures; this is referred to as chilling injury (CI) [4]. Pericarp browning is regarded as the most characteristic CI symptom in chilled litchi fruit and may be the result of cellular de-compartmentalization triggered by membrane spoilage under chilling stress, where the adequate contact of oxidative enzymes and phenol substrates yields brown insoluble pigments [5].

Although some safe strategies (such as chitosan-coating [6], modified atmosphere packing [7,8], combined applications of organic acid dipping and foil wrapping [9], L-cysteine application [10], kojic acid treatment [11], and methionine solution immersion [12]) have been demonstrated to ameliorate the chilling stress-induced browning of litchi fruit, there is still an imperative requirement to explore more methods that can reliably enhance the cold tolerance of litchi fruit. 

Melatonin (N-acetyl-5-methoxytryptamine, MLT) is a ubiquitous bioactive molecule with multiple functions in nature [13]. In higher plants, MLT is extensively distributed in almost all tissues and organs and plays important roles in multifarious physiological processes, including seed germination, floral development, photosynthesis efficiency, maturation and senescence, osmotic adjustment and resistance to numerous environmental stresses [13]. In addition to having plant growth regulator-like functions, MLT has been identified as a powerful quencher of free radicals and as an activator of the antioxidant system, contributing to the avoidance of cumulative oxidative damage to the cell membrane and cytoplasm [14,15]. Due to its natural attributes, MLT has been developed as a nutritive supplement that exhibits multiple healthcare functions, especially in improving sleep quality, protecting against aging, and modulating biological rhythms [16,17,18].

Recently, the application of MLT has displayed better performance in abating CI through the improvement in cold tolerance in several harvested fruits, including peaches [19,20], tomatoes [21,22], and pomegranates [23,24]. MLT-conferred protection against chilling stress in these crops involves the inhibition of membrane lipid peroxidation, the reinforcement of cell wall structure, activated phenol metabolism, and promotion of the biosynthesis of osmotic-adjusting compounds. In a previous study, we noticed that exogenous treatment with MLT at 400 μM effectively delayed the evolution of pericarp browning in ‘Ziniangxi’ litchi fruit during natural senescence at ambient temperature (25 °C), which was attributed to suppressed phenolic oxidation, enhanced enzymatic and nonenzymatic antioxidant activities, and the improved repair capability of oxidative-damaged proteins via the up-regulation of the expression of the genes encoding methionine sulfoxide reductases [25]. However, to our knowledge, there is no information regarding the impact of MLT on CI in litchi fruit under chilling stress and the underlying regulatory mechanisms.

Increased amounts of evidence have indicated that the maintenance of cellular energy status and the accumulation of osmolyte proline can account for the increased cold tolerance in a variety of harvested crop species [26,27]. Our objective, therefore, was to determine the impacts of MLT on CI in litchi fruit in relation to energy and proline metabolism under ambient storage conditions after removal from refrigeration.

## 2. Materials and Methods

### 2.1. Fruit Materials and Treatments

Litchi (*L. chinensis* Sonn. cv Baitangying) fruit at commercial maturity were manually collected from a farm in a suburb of Haikou city, China. The fruit were packed into foam boxes and transported to the postharvest facility of Hainan University within 1 h via a cargo van. Fruit with a uniform size and free of visual blemishes were chosen and then disinfected with 0.5% NaClO for 30 s. After washing with deionized (di) H_2_O and drying, the fruit were divided into two groups with 540 fruit per group. One group was subdivided into nine equal batches, and then each batch of fruit (60) was dipped in 400 μM MLT (Solarbio Science and Technology Co. Ltd., Beijing, China) solution prepared with 10 L of diH_2_O at 25 °C for 20 min; this treatment process was operated under low-light conditions to avoid the decomposition of MLT [25]. Another group was dipped in diH_2_O using the same procedure, which served as a control. The optimal MLT concentration of 400 μM was used based on the results of preliminary experiments using 0, 50, 100, 200, 400 and 800 μM. After dipping with the MLT solution or diH_2_O, the fruit were incubated at room temperature for 4 h to allow the adequate absorbance of MLT. The two treatment groups of fruit were packed in polyethylene bags (size: 200 mm × 150 mm; 0.03 mm thickness; nine perforations of 8 mm in diameter were provided to each side of the bag; each treatment contained 36 bags, with 15 fruit per bag) and subsequently refrigerated at 5 °C under 85–90% relative humidity (RH) for 15 d. The refrigeration conditions and packing method effectively avoided excessive water loss and anaerobic respiration. After refrigeration, the fruit were removed and stored at 25 °C and 85–90% RH to simulate shelf conditions. During storage under ambient conditions, the CI index, pericarp color and relative electrical conductivity (REC) were measured at 5 h intervals. Moreover, pericarp tissues were sampled and crushed into small pieces in liquid nitrogen before being packed, after which they were held at −80 °C for further analysis of their biochemical parameters. Each treatment for each parameter was set up in a triplicate assay. Ninety fruit from six bags in each treatment were used for the non-destructive investigation of the CI index and pericarp color, in which 30 fruit from two bags were considered as one replicate. REC was destructively analyzed using 45 fruit from three bags per treatment at each time point, with 15 fruit from each bag serving as one replicate. Meanwhile, another 45 fruit from three bags per treatment at each time point (15 fruit per replicate) were processed into frozen samples as mentioned above.

### 2.2. Determination of the CI Index

CI severity was assessed based on the degree of litchi pericarp browning, which was classified into four categories of severity [28]. The CI index was equivalent to the browning index, which was calculated by a formula [28,29], where the CI index = Σ (CI class × the number of fruit in each class)/(4 × total number of fruit in each treatment).

### 2.3. Determination of Pericarp Color

Determination of the pericarp color was based on the CIE L* a* b* color system using a Minolta CR-400 colorimeter (Konica Minolta Sensing, Inc., Osaka, Japan). The values of L*, a*, b* rankings from low to high, represent the transition from dark to bright, green to red, and blue to yellow, respectively. Two measurements were executed at two symmetric points of the equatorial axis of each fruit.

### 2.4. Determination of Anthocyanin Content

Anthocyanin content was measured following the method of Zhang et al. [30]. One gram of thawed pericarp pieces was soaked in 15 mL of 0.1 M HCl and rinsed for 24 h in a shaking bath at 25 °C. The extracts were then transferred to two tubes, with 0.5 mL extract for each tube. A total of 2.5 mL of KCl-HCl buffer (0.4 M, pH 1.0) was added to one tube, and 2.5 mL of citric acid-K_2_HPO_4_ buffer (0.4 M, pH 5.0) was added to the other tube. After fully mixing with a vortex mixer, the absorbance of the two mixtures was read at 510 nm. The anthocyanin content was calculated according to a formula described by Zhang et al. [30] and was expressed as g kg^−1^ fresh weight (FW).

### 2.5. Determination of REC

Forty-five pericarp discs (8 mm diameter per disc) derived from 15 fruit of each replicate were used to measure REC using a conductivity meter following our previous method [31]. The REC was expressed as a percentage (%) that represents the ratio of conductivity after incubation for 30 min at 25 °C to total conductivity after heating at 100 °C.

### 2.6. Determination of Malondialdehyde (MDA) Content

MDA was measured based on the thiobarbituric acid (TBA) assay, during which MDA reacts with TBA to generate a reddish-brown substance that is maximally absorbed at a wavelength of 532 nm. Specific measurement procedures were implemented using 1 g of pericarp tissues according to the report by Duan et al. [32]. The MDA content was expressed as μmol kg^−1^ FW.

### 2.7. Analysis of Energy Status

Two grams of pericarp tissues were used to determine the contents of adenosine triphosphate (ATP), adenosine diphosphate (ADP) and adenosine monophosphate (AMP) via high-performance liquid chromatography with reference to the procedures described by Liu et al. [33]. The ATP, ADP, and AMP contents were expressed as mg kg^−1^ FW. Energy charge (EC) was calculated as EC = ([ATP] + 1/2 × [ADP])/([ATP] + [ADP] + AMP]).

### 2.8. Assay of Energy Metabolism-Related Enzyme Activities

Mitochondrial H^+^-ATPase (EC 3.6.1.35), Ca^2+^-ATPase (EC 3.6.1.3), succinate dehydrogenase (SDH, EC 1.3.99.1), and cytochrome C oxidase (CCO, EC 1.9.3.1) activities were determined using 1.5 g of pericarp tissues according to the methods of Jin et al. [34]. The activities of the four enzymes were expressed as Units (U) kg^−1^ FW. One U of H^+^-ATPase and Ca^2+^-ATPase activities was defined as the enzyme amount needed to catalyze the generation of 1 nmol phosphorus per min at 660 nm, while the definition of one U of SDH and CCO activities indicates an increase of 0.01 in absorbance per minute at 660 and 510 nm, respectively.

### 2.9. Assay of Proline Metabolism

The proline content was determined based on the acid ninhydrin method, in reference to the detailed procedure reported by Zhang et al. [35]. The proline content was expressed as mg kg^−1^ FW.

The extraction and activity determination of ornithine-δ-aminotransferase (OAT, EC 2.6.1.13) were performed according to the methods of Roosens et al. [36] and Kim et al. [37] using 1 g of pericarp tissues. One U of OAT activity was defined as the amount of enzyme catalyzing the generation of 0.01 μmol of Δ^1^-pyrroline-5-carboxylic acid (P5C) per minute. The OAT activity was expressed as U kg^−1^ FW.

P5C synthase (P5CS, EC 2.7.2.11) and proline dehydrogenase (PDH, EC 1.5.99.8) activities were determined following the methods of Shang et al. [38] using 1 g of pericarp tissues. One U of P5CS and PDH activities was defined as the amount of enzyme that causes a change in the absorbance of 0.001 at 340 nm per minute in their respective reaction systems, and the specific activity of each enzyme was expressed as U kg^−1^ FW.

### 2.10. Statistical Analysis

All the data are presented as the means ± standard errors (SEs). The data were analyzed by independent sample *t*-test using the IBM SPSS statistical 22.0 software (SPSS Inc., Chicago, IL, USA). Significant differences in the means between groups at the same time point were compared. Asterisks (* and **) were used to represent significant differences at *p* < 0.05 and *p* < 0.01, respectively.

## 3. Results

### 3.1. CI Index of the Pericarp

**Figure 1:**
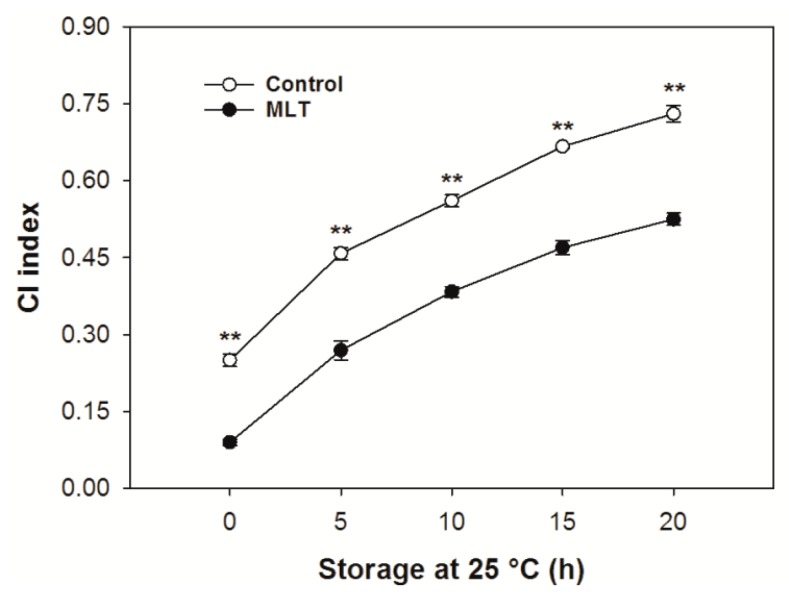
Chilling injury (CI) index of the control and melatonin (MLT)-treated litchi (cv. Baitangying) fruit during storage at 25 °C after 15 d of refrigeration at 5 °C. The bars represent the SEs of the means (*n* = 3). The asterisks indicate that the values are significantly different between groups at the same time point (* *p* < 0.05, ** *p* < 0.01).

Control and MLT-treated litchi fruit developed different degrees of CI after 15 d of refrigeration at 5 °C, but the MLT-treated fruit had a lower CI index than the control fruit at the end of refrigeration (0 h) (Figure 1). The CI severity of the fruit, regardless of treatment, gradually increased during 20 h of storage at 25 °C after the removal from low temperature (Figure 1). However, an extremely significant (*p* < 0.01) lower CI index was detected in the MLT-treated fruit throughout the 20 h of storage at 25 °C compared to that of the untreated fruit (Figure 1).

### 3.2. Pericarp Color

**Figure 2:**
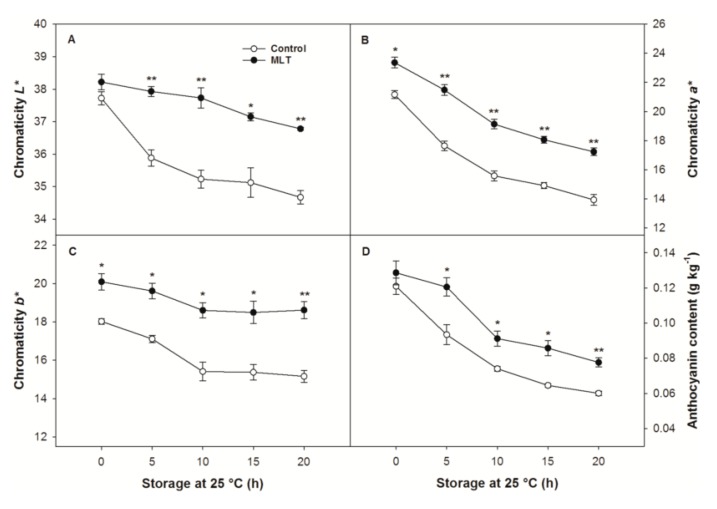
(**A**) chromaticity L*, (**B**) chromaticity a*, (**C**) chromaticity b* values, and (**D**) anthocyanin content of the control and MLT-treated litchi (cv. Baitangying) fruit during storage at 25 °C after 15 d of refrigeration at 5 °C. The bars represent the SEs of the means (*n* = 3). The asterisks indicate that the values are significantly different between groups at the same time point (* *p* < 0.05, ** *p* < 0.01).

Compared to the control treatment, the pericarp of the litchi fruit with the MLT treatment maintained higher chromaticity a* and b* values at the end of refrigeration (0 h), but chromaticity L* was not affected by MLT treatment at 0 h (Figure 2A–C). The chromaticity L*, a*, b* values in both the control and MLT-treated fruit dropped overall with an increased storage duration at 25 °C after refrigeration (Figure 2A–C). Declines in these three color parameters were inhibited by MLT treatment, in which the chromaticity L*, a*, b* values in the fruit receiving MLT were, on average, 6.2%, 22.4%, and 19.6% higher than those in the control fruit during 5–20 h of storage at 25 °C after refrigeration (Figure 2A–C). Anthocyanin, the pigment contributing to red color, presented a continuous decline in content in parallel with changes in the chromaticity a* (redness) of the fruit, regardless of treatment (Figure 2B,D). The application of MLT to the litchi fruit, however, significantly slowed the decrease in pericarp anthocyanin content in the litchi fruit within 5–20 h of storage (Figure 2D).

### 3.3. REC and MDA Content

**Figure 3:**
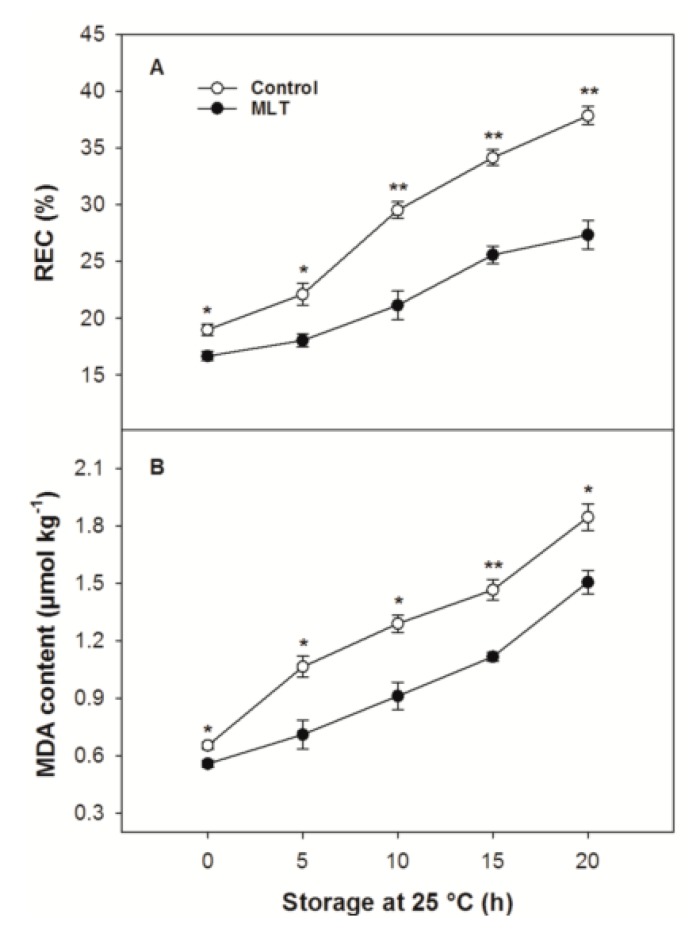
(**A**) relative electrical conductivity (REC) and (**B**) malondialdehyde (MDA) content of the control and MLT-treated litchi (cv. Baitangying) fruit during storage at 25 °C after 15 d of refrigeration at 5 °C. The bars represent the SEs of the means (*n* = 3). The asterisks indicate that the values are significantly different between groups at the same time point (* *p* < 0.05, ** *p* < 0.01).

The control and MLT-treated fruit differed in both their REC and MDA content at the end of refrigeration (0 h) (Figure 3A,B). REC and MDA content in the control fruit showed 2- and 2.8-fold increases after storage for 20 h at 25 °C, respectively (Figure 3A,B). MLT treatment strongly repressed the increases in REC and MDA levels, whose parameter values were, on average, 23.7% and 24% lower than those of the control fruit throughout storage at 25 °C (Figure 3A,B).

### 3.4. ATP, ADP and AMP Contents and EC

**Figure 4:**
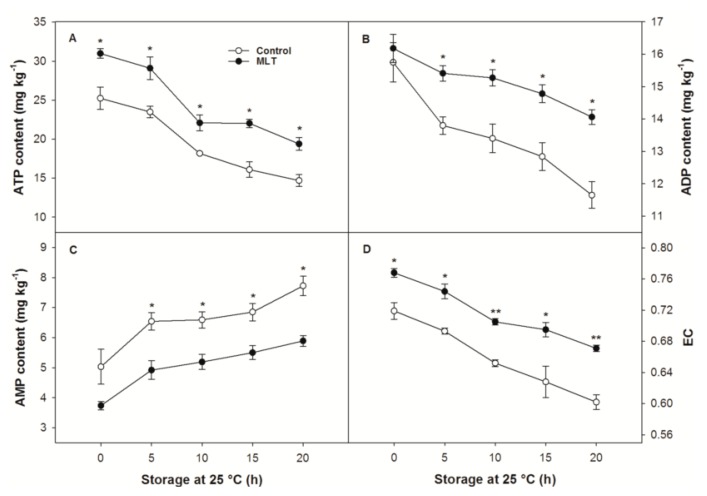
(**A**) adenosine triphosphate (ATP), (**B**) adenosine diphosphate (ADP), and (**C**) adenosine monophosphate (AMP) contents, and (**D**) energy charge (EC) of the control and MLT-treated litchi (cv. Baitangying) fruit during storage at 25 °C after 15 d of refrigeration at 5 °C. The bars represent the SEs of the means (*n* = 3). The asterisks indicate that the values are significantly different between groups at the same time point (* *p* < 0.05, ** *p* < 0.01).

There were significant differences in ATP content and EC, but not in ADP and AMP contents, between the control and MLT-treated fruit at the end of refrigeration (0 h) (Figure 4). The ATP and ADP contents in the fruit, irrespective of the treatments, gradually declined as storage duration progressed, but slower rates of decline were observed for MLT-treated fruit than for the control fruit (Figure 4A,B). The AMP content in the control fruit continuously rose throughout storage (Figure 4C). MLT treatment resulted in lower AMP content than that in the control from 5 to 20 h of storage (Figure 4C). Both the control and MLT-treated fruit exhibited approximately linear declines in EC during storage, but higher EC values were observed in the latter (Figure 4D).

### 3.5. Enzymatic Activity Related to Energy Metabolism

**Figure 5:**
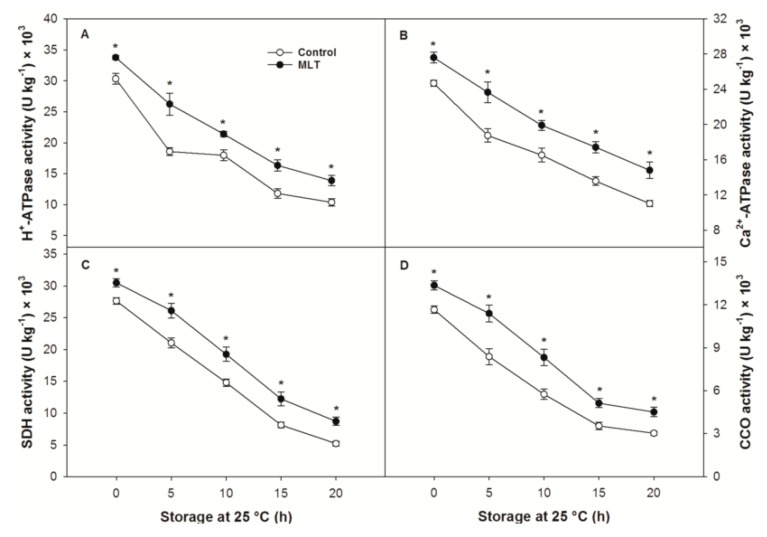
(**A**) H^+^-ATPase, (**B**) Ca^2+^-ATPase, (**C**) succinate dehydrogenase (SDH), and (**D**) cytochrome C oxidase activities of the control and MLT-treated litchi (cv. Baitangying) fruit during storage at 25 °C after 15 d of refrigeration at 5 °C. The bars represent the SEs of the means (*n* = 3). The asterisks indicate that the values are significantly different between groups at the same time point (* *p* < 0.05, ** *p* < 0.01).

There were significant differences in the activities of H^+^-ATPase, Ca^2+^-ATPase, SDH and CCO between the control and MLT treatments at the end of refrigeration (Figure 5A–D). After removal from refrigeration, the activities of these four enzymes sharply dropped with the progression of storage (Figure 5A–D). The declining process of enzyme activities was slowed by treatment with MLT, and significantly higher activity values were noted in the MLT-treated fruit throughout storage (Figure 5A–D).

### 3.6. The Proline Metabolic Pathway

**Figure 6:**
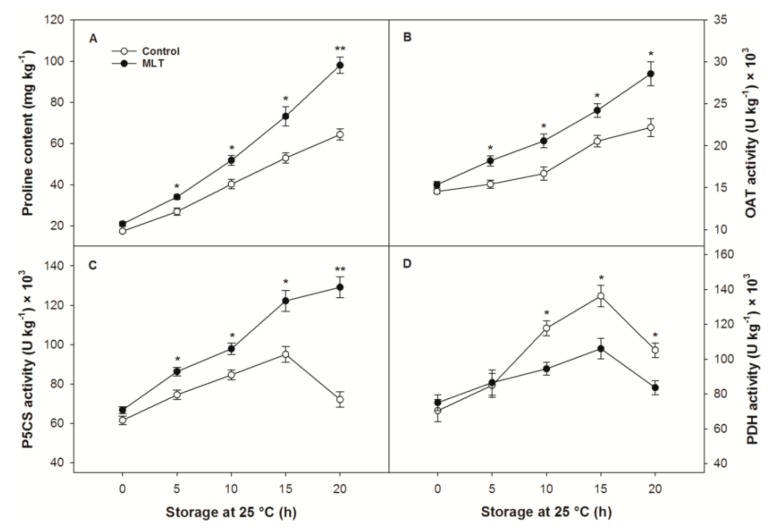
(**A**) proline content and activities of (**B**) ornithine-δ-aminotransferase (OAT), (**C**) Δ^1^-pyrroline-5-carboxylic acid synthase (P5CS) and (**D**) proline dehydrogenase (PDH) of the control and MLT-treated litchi (cv. Baitangying) fruit during storage at 25 °C after 15 d of refrigeration at 5 °C. The bars represent the SEs of the means (*n* = 3). The asterisks indicate that the values are significantly different between groups at the same time point (* *p* < 0.05, ** *p* < 0.01).

The proline content and activities of its related metabolic enzymes, including P5CS, OAT, and PDH, in the control and MLT-treated fruit did not vary after refrigeration (Figure 6A–D). During storage at 25 °C, the control fruit displayed a steady accumulation of proline that was induced more strongly by MLT treatment during 5–20 h of storage (Figure 6A). The OAT activity in the control and MLT-treated fruit exhibited similar tendencies to those recorded for proline content, with significant differences occurring from 5 to 20 h of storage (Figure 6B). The P5CS and PDH activities in the control fruit exhibited both increasing and declining trends during storage (Figure 6C,D). MLT treatment promoted the enhancement of P5CS activity while reducing PDH activity during most of the storage period (Figure 6C,D).

## 4. Discussion

Rapid pericarp browning after removal from low-temperature conditions is a major factor limiting the application of refrigeration to litchis [3]. As an important antioxidant and a modulator of multiple physiological behaviors in planta, MLT was applied to harvested ‘Baitangying’ litchi fruit to assess its capability to regulate chilling tolerance in fruit under shelf conditions at 25 °C after 15 d of refrigeration at 5 °C. The results showed that 400 μM MLT treatment markedly retarded the development of CI and prevented pericarp discoloration, as shown by the higher chromacity L*, a*, b* values and anthocyanin content in the MLT-treated fruit. Similar CI alleviation and suppressed quality deterioration due to MLT treatment were reported in tomatoes [21,22], pomegranates [23] and peaches [39].

Severe chilling stress may trigger the transition from the liquid-crystalline phase of membrane lipids to the solid-gel phase, leading to the irreversible destruction of membrane integrity and the accelerated leakage of ions. Accumulating evidence suggests that a loss of membrane integrity may provoke subcellular de-compartmentalization, resulting in enzymatic browning catalyzed by peroxidase and polyphenol oxidase in postharvest fruits [32]. This damage to membrane integrity is reflected by changes in membrane permeability, which can be determined by the REC [35]. The process of membrane disruption under chilling stress involves lipid peroxidation initiated by the chain reactions of free radicals [40]. MDA, the final decomposition product of lipid peroxidation, has been employed as an indicator of the degree of oxidative stress in plants [26]. In the current study, REC and MDA content continuously increased with the progression of CI development in litchi fruit under ambient shelf conditions after cold storage, whereas MLT treatment strongly repressed the increases in both parameters, suggesting that the prevention of CI afforded by MLT might be involved in reducing the oxidative destruction of membranes. Corroborating our results, Jannatizadeh et al. [21] observed that MLT treatment inhibited membrane phospholipid degradation and maintained the unsaturation degree of fatty acids, consequently contributing to the preservation of membrane integrity and the amelioration of CI in tomato fruit during refrigeration at 4 °C.

Accumulating evidence has recently indicated that cellular energy status is a pivotal factor regulating senescence and the response to various environmental stresses [27]. Liu et al. [4] noted that chilling stress-induced litchi pericarp browning involves reduced cellular energy levels and increased activities of phospholipid-metabolizing enzymes, indicating that insufficient energy supplies could be accountable for restricting the synthesis of phospholipids and hastening their hydrolysis. By contrast, exogenous ATP treatment could promote the generation of endogenous ATP in pear fruit, which could in turn strengthen the membrane structure and thus prevent CI occurrence under shelf conditions at ambient temperature after refrigeration [41]. Moreover, various postharvest approaches to elevate energy levels have promoted the improvement in cold tolerance in fruits, as demonstrated in low-temperature-conditioned loquats [42], 1-methylcyclopropene-treated pears [43], H_2_S-treated bananas [44], and glycine betaine (GB)-treated papayas [40]. In the current results, MLT treatment markedly delayed declines in the ATP levels and EC in litchi fruit throughout storage at 25 °C following refrigeration, which might be of benefit for maintaining membrane integrity and suppressing CI. Similar observations have been reported in MLT-treated tomato fruit during cold storage [21].

The cellular energy level in plant cells is generally modulated by the mitochondrial enzymes associated with energy metabolism, such as H^+^-ATPase, Ca^2+^-ATPase, SDH, and CCO [44,45]. Among these enzymes, H^+^-ATPase can build up transmembrane potential by transporting H^+^ out of cells, coupled with the hydrolysis of ATP, thereby causing the release of energy and thus enabling endoergic reactions in cells [46]. Ca^2+^-ATPase, a Ca^2+^ pump, catalyzes the hydrolysis of ATP into ADP and phosphate, along with the release of energy that can force superfluous Ca^2+^ into the extracellular matrix, thus stabilizing intracellular Ca^2+^ concentration and maintaining physiological functions in plant cells [28]. A reduction in both ATPase activities has been correlated to a loss of membrane integrity, suggesting that decreasing membrane energization can weaken resistance to stresses and initiate cell dysfunction [47]. SDH is one of the central junctions connecting oxidative phosphorylation and electron transport and is considered a marker for evaluating the efficiency of the tricarboxylic acid cycle, in which SDH oxidizes succinate to fumarate while generating ATP [48]. CCO is the terminal enzyme in the mitochondrial respiratory chain and can directly pass electrons of the respiratory substrate to dioxygen, coupled with the generation of ATP via oxidative phosphorylation [49]. Inactivated SDH and CCO inevitably hinder the synthesis and utilization of energy, resulting in physiological disorders in harvested fruit [40]. In the current work, the decreasing activities of H^+^-ATPase, Ca^2+^-ATPase, SDH and CCO, in parallel with the progression of CI in litchi fruit, were slowed by MLT treatment, indicating that MLT might enhance cold tolerance via the modulation in energy status associated with mitochondrial energy metabolism. A similar CI control in association with activated energy metabolism was also observed in MLT-treated tomato fruit refrigerated at 4 °C [21].

Proline, a nonpolar amino acid, has been recognized as a crucial osmolyte that can stabilize inorganic ion concentrations and improve the water-holding capacity of plant cells, thereby contributing to the balance of cell osmotic potential [50]. Proline also plays an active role in stabilizing biomacromolecules and maintaining cellular redox homeostasis by removing redundant reactive oxygen species [51]. The accumulation of proline commonly occurs when plants are exposed to abiotic stress, which is conducive to protecting tissues against stress-induced oxidative damage [52]. Proline in plants can be synthesized by the glutamate and ornithine pathways, each of which is involved in the catalysis of a key enzyme (P5CS and OAT, respectively) [53]. PDH is the rate-limiting enzyme responsible for catalyzing proline decomposition [51]. The mode of proline accumulation rests in the dynamic equilibrium between its synthesis and degradation, which is associated with changes in the above-mentioned proline metabolic enzyme activities [54]. The present results exhibited a higher accumulation of proline accompanied by reduced CI in litchi fruit receiving MLT compared to the result for untreated fruit during storage after refrigeration, which might be the result of elevated activities of OAT and P5CS, as well as suppressed PDH activity. The results indicate that MLT could enhance the resistance of litchi fruit to chilling stress by inducing the synthesis of proline. Similarly, the improvement in chilling tolerance in relation to the promoted synthesis and limited degradation of proline under the modulation of proline metabolic enzymes has also been found in methyl jasmonate-treated loquats [55], oxalate-treated mangoes [56], nitric oxide-treated bananas [57], 24-epibrassinolide-treated peaches [52], MLT-treated tomatoes [22] and GB-treated pears [54].

## 5. Conclusions

In conclusion, the present results demonstrated that pretreatment with 400 μM MLT effectively reduced CI occurrence and slowed the discoloration in the ‘Baitangying’ litchi fruit stored at ambient conditions after removal from refrigeration. The protection afforded by MLT might be attributed to the reduced damage to the cell membrane, which was associated with the activated energy and proline metabolism. Hence, MLT treatment could be a promising approach to improve cold tolerance and alleviate CI in harvested litchi fruit.
